# Microbial Primer: Ancientbiotics – making modern antimicrobials from historical infection remedies

**DOI:** 10.1099/mic.0.001642

**Published:** 2026-01-29

**Authors:** Freya Harrison, Oluwatosin Q. Orababa

**Affiliations:** 1 School of Life Sciences, University of Warwick, Coventry, UK

**Keywords:** anti-bacterial agents, antibiotics, antimicrobials, interdisciplinary, natural product, treatment

## Abstract

The modern antibiotic era began in the early twentieth century, but humans have long used materials from the natural world to attempt to treat the symptoms of infection. In this primer, we will discuss the rationale for attempting to reconstruct historical infection remedies in order to assess their antimicrobial activity and how this approach could aid the discovery of molecular cocktails with potential for development into novel treatments for infection.

## Why should microbiologists be interested in historical remedies?

We are entering a post-antibiotic era, with a stalled antibiotic discovery pipeline and ever-rising rates of drug-resistant infection. In the hunt for new antimicrobial drug candidates, microbiologists must cast our net beyond the soil-dwelling microbes that have provided most of our current antibiotic armoury. In the millennia before the revolutionary discoveries of carbolic acid, sulphonamides and penicillin in the late nineteenth and early twentieth centuries, people used a wide range of natural materials to attempt to treat the pathogenic microbes which have been our constant companions. In this primer, we will explore how preparations of natural products (NPs) used in pre-modern medicine may be reconstructed in the lab and the potential of these *ancientbiotics* (Box 1: Glossary) to reveal combinations of molecules worth exploring for their potential to prevent and treat microbial infection.

Box 1. **Glossary**

**Ancientbiotics**

Pre-modern antimicrobial preparations. In strict terms, ‘ancient’ history ends around the year 500 CE, but we and others have used this term as shorthand for historical remedies regardless of the period of origin.

**Ethnopharmacology**

The study of traditional or folk medicines used by particular cultural or ethnic groups, and their societal context.

**Experimental archaeology**

A sub-discipline of archaeology that attempts to test hypotheses about archaeological materials, artefacts, practices or processes by attempting to replicate them in controlled conditions.

**Natural product**

In medicinal chemistry, this term refers to a chemical compound made by a living organism, that is a secondary metabolite. This definition usually refers only to small molecules (e.g. antibiotics). In a broader sense, the term may additionally include proteins (e.g. antimicrobial peptides) and mixtures of substances produced by organisms (e.g. whole botanical extracts, honey).

**Potentiator**

A molecule that is not itself an active antimicrobial but which enhances the antimicrobial activity of another molecule. Examples include β-lactamase inhibitors, efflux pump inhibitors or biofilm matrix-degrading agents.

**Synergy**

Two drugs are said to act additively if the effect of combination treatment (e.g. reduction in viable pathogen load) is equal to the sum of the effects of each drug used individually. If the effect of combination treatment is significantly greater than the sum of their individual effects, then the drugs are said to act synergistically.

**Fig. 1.**
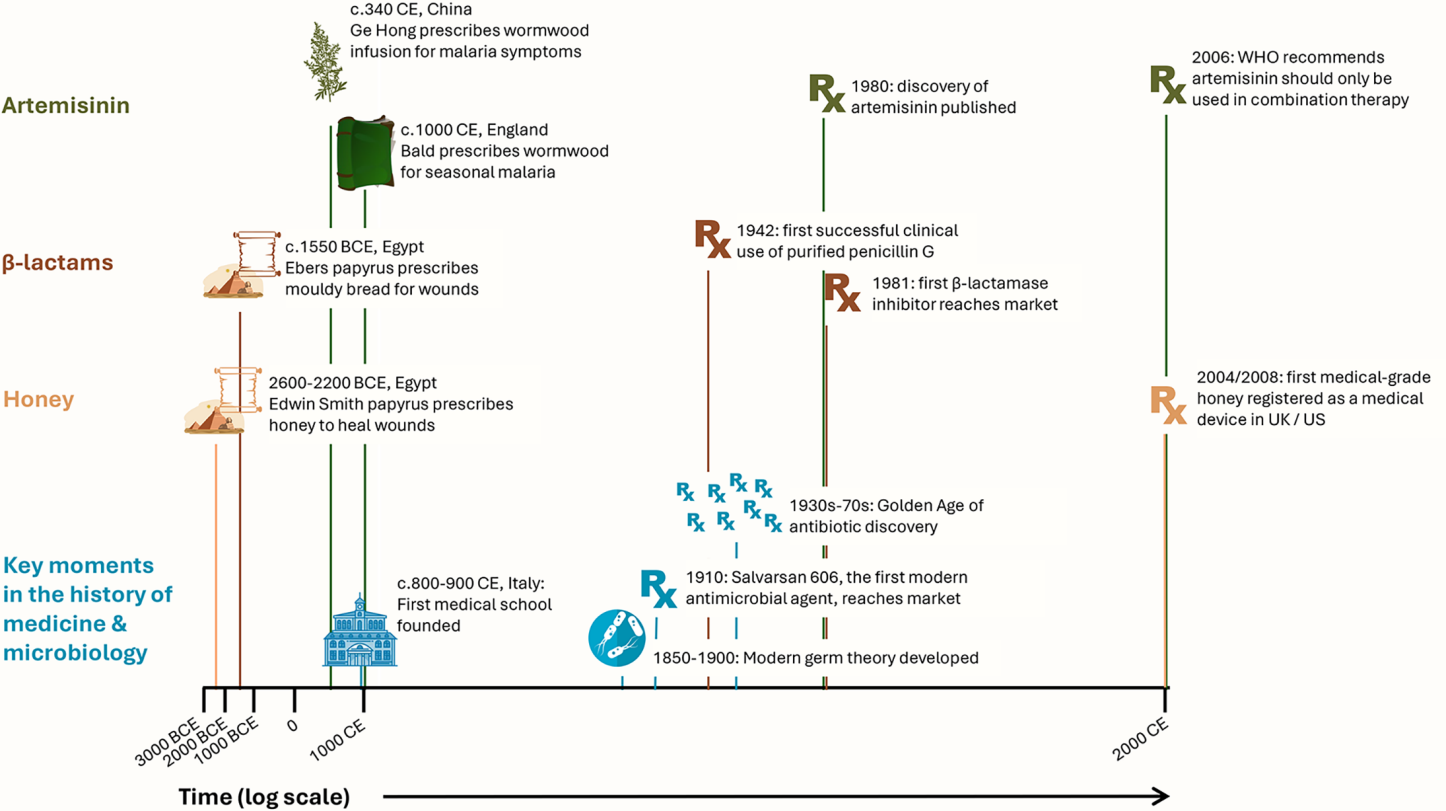
Key moments in the history of human use of three NPs discussed in this primer: artemisinin, honey and β-lactam antibiotics, and key moments in the history of microbiology. Icons: Creazilla open licence.

Attempts to treat infection or its symptoms (inflammation, fever etc.) in the past relied upon a very different understanding of the human body, and very different concepts of disease aetiology, than we now possess. Nevertheless, in their attempts to use the tools and materials available to them to alleviate the symptoms of disease, our ancestors did discover a range of NPs that worked to some degree or another (Fig. 1). Some of these treatments developed into well-evidenced pharmaceuticals that are still used today. Among the earliest recorded medical texts, the ancient Egyptian Ebers papyrus (written circa 1550 BCE) may prefigure the discovery of penicillin by prescribing the use of mouldy bread as a wound dressing. Other examples relevant to treating infection or inflammation include honey (used in modern wound care to manage infection and promote healing); aspirin (a synthetically modified derivative of salicylic acid, which was traditionally obtained from willow bark by chewing or by preparing a liquid extract); acetic acid (traditionally used in the form of vinegar and now used to treat burn wound infections); artemisinin (an antimalarial compound derived from *Artemisia* species, whose use in drinks for treating symptoms consistent with malaria was documented in medical texts from fourth-century China and tenth-/eleventh-century England) and even an early form of vaccination (variolation, i.e. deliberate infection with cowpox, was a documented preventative measure against smallpox in China, India, Africa, Europe and the Middle East in the sixteenth to eighteenth centuries). More broadly, a global survey of 1881 drugs approved for clinical use between 1981 and 2019 found that while fully synthetic molecules and vaccines accounted for approximately one-third of all approved agents, the remaining two-thirds comprised NPs, synthetically modified NPs, mimics of NPs, synthetic molecules inspired by NPs, botanical mixtures and biological macromolecules (see Newman and Cragg in *Further Reading*). Therefore, it seems sensible to continue to explore NPs used in historical and traditional medicine to find new agents to prevent and treat microbial infection (see also Quave and Tu in *Further Reading*).

However, just as antibiotic discovery research suffers from the problem of rediscovering the same compounds, over the last few decades NP research has largely uncovered new NPs that are structurally very similar to those already known about. The rate of discovery of novel structures appears to be flatlining (see Pye *et al*. in *Further Reading*). One potential way to unlock the discovery of more diverse and more biologically active NPs is for microbiologists and chemists to work with historians and historical manuscript specialists to more thoroughly understand how natural materials were processed into remedies and assigned to treat particular symptoms. This approach could yield novel compounds that are not easily extracted from organic material by standard laboratory procedures. Successful artemisinin extraction famously required steeping plant material in cold water, as specified by the fourth-century author of a Chinese remedy for ‘intermittent fevers’, rather than modern extraction methods using organic solvents. This approach could also lead to the discovery of combinations of NPs that *synergize* or *potentiate* one another to create a cocktail whose biological activity is greater than the sum of each of its constituent molecules alone.

## The importance of mixtures in ancientbiotics

**Fig. 2.**
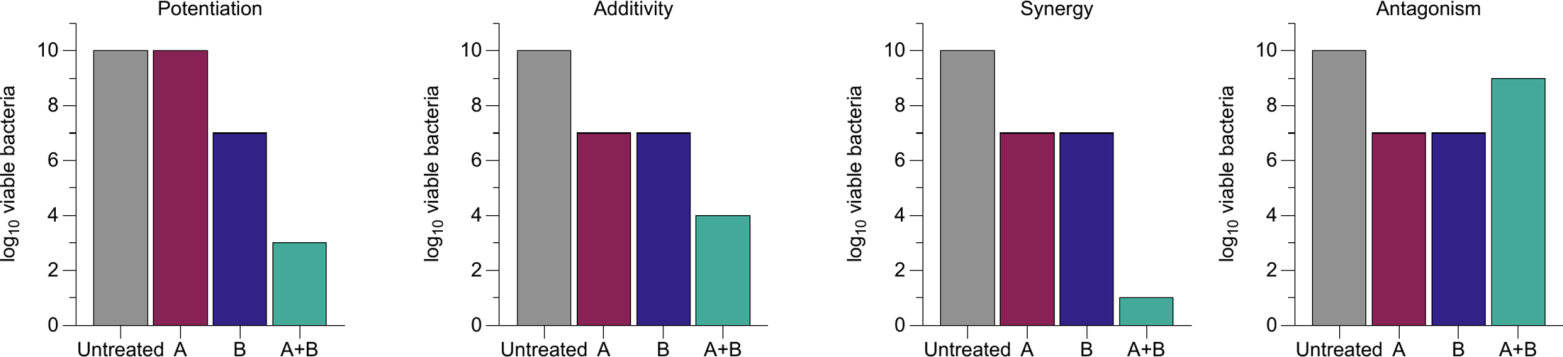
Four examples of interactions between hypothetical molecules A and B, when applied in combination to bacterial culture. Potentiation: drug A has no direct effect on bacterial viability, but it enhances the activity of a bactericidal drug, B. Additivity: both A and B have bactericidal effects, and when used in combination, the combined effect is the same as the sum of the effect of each drug used alone. Synergy: both A and B have bactericidal effects, and when used in combination, the combined effect is greater than the sum of the effect of each drug used alone. Note that depending on the mechanisms of action of A and B, and their dose–response relationships, the exact combined effect predicted under additivity can vary. This is explored in detail by Duarte and Vale (see *Further Reading*). Antagonism: both A and B have bactericidal effects, but when used in combination, the combined effect is less than the sum of the effect of each drug used alone.

Many modern antimicrobials have a single mechanism of action or cellular target. However, agents with multiple targets, or combined therapy with two or more agents with different targets, can increase the efficacy of treatments and make it harder for pathogens to evolve resistance (see Feng *et al*. and Bognár *et al*. in *Further Reading*). Approaches that involve the combination of antibiotics with *potentiators *(compounds with no significant antimicrobial effect that improve the activity of antimicrobials) have been successfully implemented in the clinic. A common example of this is the combination of β-lactam antibiotics and β-lactamase inhibitors. Multiple antibiotics have also been combined to treat highly resistant infections, and these may have useful additive or *synergistic *antibacterial activity (Fig. 2). For example, one approved treatment for *Mycobacterium tuberculosis* is the combination of ethambutol, isoniazid, pyrazinamide and rifampicin; and Neosporin^TM^ wound ointment contains neomycin, polymyxin B and bacitracin.

Oftentimes, traditional remedies include combinations of more than one material – usually herbal. Each of these materials may contain hundreds of bioactive compounds, some of which act additively or synergistically to confer improved bioactivity or broad-spectrum activities on these treatment mixtures. The conventional drug discovery approach to solving this complexity challenge involves the identification and purification of a major active compound in the mixture. However, this masks the overall activity of the mixture, as some additives or synergists will be lost. For example, the use of *Artemisia annua* whole plant slowed down resistance evolution in *Plasmodium* species compared to purified artemisinin. A good example of a complex multipartite ingredient that has been used to treat infections for centuries, and which cannot be replaced by a single compound, is honey. The antibacterial efficacy of honey is associated with the presence of methylglyoxal or peroxidase, phenolics and a high osmotic potential. This complexity in bioactive constituents has led to no reported resistance evolution against honey. Similarly, optimal antibacterial activity of wine (another medicinal medieval ingredient) is associated with the interaction between its components (pH, polyphenols, organic acids and alcohol). Furthermore, optimal antibiofilm efficacy of a complex medieval remedy investigated by our team, Bald’s eyesalve, requires all its ingredients (onion, garlic, wine and bovine bile). It is also difficult to evolve resistance against this complex remedy. Taken together, there is compelling evidence that mixtures of bioactive NPs found in ancientbiotics could be explored further to develop modernized treatments for infections and have a reduced risk of resistance evolution.

Despite the promising potential of using complex ancientbiotic mixtures to treat infection, the issue of standardization and reproducibility of these mixtures needs to be addressed. To use mixtures in their original extract state while maintaining reproducibility, selected compounds with the highest activity contributions can be identified and quantified as markers for standardization (e.g. manuka honey is classified by methylglyoxal content). Alternatively, these selected compounds could be purified and made into a well-defined cocktail formulation, which retains most of the activity of the original mixture. This approach requires a great understanding of the interaction between the major bioactive compounds in the mixture. Even though it might not totally represent the activity of the original mixture, it could at least retain some of its complexity and reduce the risk of resistance due to the collective mechanism of the constituting compounds. Combining multiple compounds from a complex mixture is likely to be particularly important for complex infections involving biofilms because a multicomponent compound mixture could attack different aspects of a biofilm that contribute to its high antibiotic tolerance (e.g. cell viability, matrix integrity and cell–cell signalling).

## Defining your goals

There are several reasons why microbiologists might study ancientbiotics, and it is important to clearly define your precise motivation. On the one hand, your approach may be one of *experimental archaeology*: you are interested in how a remedy was made and how it might have really worked. You will not obtain a definitive answer to these questions (short of acquiring a time machine), but you might be able to gain some insight into historical ways of working with medical materials. In this approach, you will apply your skills as a microbiologist to help historians of science and medicine explore their field. On the other hand, your motivation may be drug discovery: you are interested in using a historical remedy or collection of remedies as a mini ‘database’ to find interesting compounds with antimicrobial activity and cocktails of compounds whose additive or synergistic interactions produce useful levels of antimicrobial activity. These questions are microbiology-led, but interdisciplinarity is still key to conducting rigorous and well-informed research. You will need to work with historians, linguists, manuscript experts and perhaps also data scientists to translate, interpret, understand and contextualize the remedy or remedies of interest. If you are exploring remedies that are still being used as traditional medicine by specific cultural or ethnic groups, then your work also intersects with *ethnopharmacology*. You may further need to employ systematic review or meta-analytic tools to assess the extent and quality of published research into the biological activity of NPs of interest. Once you have assembled your team and agreed on a common goal and terms of reference, it is time to reconstruct and test your chosen remedy.

## A toolkit for discovering promising antimicrobial combinations of NPs using historical remedies

**(a) Reconstruction of remedies**

Our research group is part of the interdisciplinary Ancientbiotics Consortium, and we recently published a case study of our experience of interdisciplinary work, which discusses the questions, challenges and synergies that arose (see Connelly *et al*. in *Further Reading*). Many of these are specifically relevant to reconstructing recipes for multi-ingredient remedies, and below we list those that are especially important for microbiologists whose aim is to identify NP cocktails with meaningful antimicrobial activity.

The fundamental question is: how accurately can and should you attempt to reconstruct your chosen remedy? When it comes to ingredient choice, issues to consider are as follows:

- How to translate words in a historical language, which may not have a single, unambiguous modern translation, e.g. what plant species is meant by a particular historical name; how many variant names or spellings refer to the same species?
- The effect of selective breeding, climate change and loss of genetic diversity on the plant varieties and plant-derived products available now versus in the period when your remedy was recorded, e.g. do modern wild or commercially grown vegetables, or modern wines, contain the same NPs, in similar concentrations, as the versions of these materials available to the people who recorded or used your selected remedy?
- Similarly, the effects of seasonality or local geography on the chemical composition of NPs.
- Where there is ambiguity in identifying ingredients, or multiple possible variations of an ingredient, how many of these alternatives is it advisable or feasible for you to compare?

To some extent, when your primary aim is drug discovery, the answers to the above questions can be informed and narrowed down by applying modern knowledge of NPs and their effects. It would be justifiable in this case to select ingredient variants that are known to contain high concentrations of NPs with demonstrated antimicrobial or anti-inflammatory activity and/or to leave out ingredients that are known to be highly toxic or irritating to eukaryotic cells and tissue.

As the example of artemisinin demonstrates, preparation methods specified in a historical remedy can be just as important as the ingredients for obtaining a bioactive product. Questions to be asked from this perspective include:

- What amount of each ingredient is required? How were ingredients measured, and is an amount even specified? Some historical texts may not state an amount, or may say something to the effect of ‘use as much as is needed’.
- Are there other missing instructions due to presumed knowledge on the part of the person who recorded the remedy? It is not uncommon for pre-modern medical texts to specify that ingredients should be prepared ‘as all doctors know’, or to refer to an extract of a natural material without specifying exactly how that extract should be made. This is an area where conversations with experts from the humanities are especially important, as they can apply a wider knowledge of medical and scientific practice from the relevant cultural context to suggest likely answers to these questions.
- How should the finished recipe be stored? Is it likely to be light- or temperature-sensitive, for instance? Can (and should) it be sterilized for later use and, if so, what methods are most likely to achieve this without altering the chemistry of the NP cocktail?

Again, if your main aim is drug discovery, it can be appropriate to apply modern knowledge to optimize recovery of promising NPs. Working with analytical chemists may be invaluable in designing your protocol for preparing a derivative of a historical remedy with the best chances of producing something worth taking into antimicrobial testing.

**(b) Antimicrobial testing media and models for predicting interactions in complex historical mixtures**

There are several factors to consider when evaluating historical remedies for their antimicrobial/antibiofilm efficacy. The first consideration is the testing model used to assess antimicrobial/antibiofilm susceptibility. The clinical translation of laboratory efficacy results of historical remedies is highly dependent on the use of appropriate media and models. Many models used in antimicrobial susceptibility testing in both research and clinical laboratories do not accurately mimic *in vivo* infection environments. This has led to inconsistencies in antimicrobial efficacy results (see Ersoy *et al*. in *Further Reading*). Consequently, studies assessing the antimicrobial efficacies of historical remedies should incorporate testing in high-validity host-mimicking media and models that are comparable to *in vivo* infection environments. This is of particular importance in antibiofilm testing. Many biofilm tests are done in 96-well plates or with the Calgary device and with standard laboratory media like Luria/lysogeny broth and tryptic soy broth. Although these models are cheap and easy to work with, they rarely provide the same environmental conditions (nutrients, pH, etc.) as found in *in vivo* infection environments. This is especially important if you wish to target chronic biofilm infections like chronic wounds or cystic fibrosis lung infections. As a result, accurate prediction of antimicrobial efficacy requires the use of the right media and models (*in vitro*, *ex vivo* or *in vivo*).

Another important consideration is the use of appropriate mathematical models for predicting compound interactions in historical mixtures. It has been previously shown that ingredients in historical remedy preparations work additively or synergistically. For instance, honey synergizes with vinegar in biofilm eradication assays in a synthetic wound model (see Harrison *et al*. in *Further Reading*). Many studies that have looked into antimicrobial compound synergies planktonically do this with the fractional inhibitory concentration index approach, while antimicrobial synergies in biofilm models are often just predicted with the highest single agent (HSA) model. These methods do not provide a comprehensive approach to synergy prediction. For instance, the HSA approach does not consider the possible additive effect between compounds and is mainly suitable for combinations where one of the agents has no antimicrobial efficacy at any of the concentrations tested (i.e. potentiator + antimicrobial mixtures, not mixtures of multiple antimicrobials). However, this is not the case for many ingredients and compounds in historical mixtures (see Caesar and Cech and Duarte and Vale in *Further Reading*).

Mathematical reference models that predict the outcome of combination treatment under the assumption of additive antimicrobial effects can be implemented here, and the observed outcome can be compared with this prediction to conclude whether the combination has additive, synergistic or antagonistic effects. It is important to consider the assumptions that different reference models make about the agents’ mechanisms of action. The agents in historical mixtures may have multiple mechanisms of action or targets, and, in many cases, the specific mechanisms of action of historical ingredients remain unknown. Hence, accurate and comprehensive prediction of antimicrobial/antibiofilm interaction requires the use of more than one synergy prediction model: if all models predict synergy, we may be reasonably confident that it exists.

## Conclusions

In this primer, we have been able to show that historical remedies hold great potential as sources of new treatments to tackle antimicrobial-resistant infections. However, there is a need for more studies that focus on interactions between multiple compounds present in these complex mixtures.

## Further reading

1. Bognár, B., Spohn, R. and Lázár, V. (2024) Drug combinations targeting antibiotic resistance. *npj Antimicrobials and Resistance* 2(1): 29.
2. Caesar, L. K. and Cech, N. B. (2019) Synergy and antagonism in natural product extracts: when 1+1 does not equal 2. *Natural Product Reports* 36(6): 869–888.
3. Connelly, E., Lee, C., Furner-Pardoe, J., del Genio, C. I. and Harrison, F. (2022) A case study of the Ancientbiotics collaboration. *Patterns* 3(12):100632.
4. Duarte, D. and Vale, N. (2022) Evaluation of synergism in drug combinations and reference models for future orientations in oncology. *Current Research in Pharmacology and Drug Discovery* 3 : 100110.
5. Ersoy, S. C., Heithoff, D. M., Barnes, L. V., Tripp, G. K., House, J. K., Marth, J. D., Smith, J. W. and Mahan, M. J. (2017) Correcting a fundamental flaw in the paradigm for antimicrobial susceptibility testing. *EBioMedicine* 20 : 173–181.
6. Feng, J., Zheng, Y., Ma, W., Ihsan, A., Hao, H., Cheng, G. and Wang, X. (2023) Multitarget antibacterial drugs: An effective strategy to combat bacterial resistance. *Pharmacology and Therapeutics* 252 : 108550.
7. Harrison, F., Blower, A., de Wolf, C. and Connelly, E. (2023) Sweet and sour synergy: exploring the antibacterial and antibiofilm activity of acetic acid and vinegar combined with medical-grade honeys. *Microbiology* 169(7).
8. Newman, D. J. and Cragg, G. M. (2020) Natural products as sources of new drugs over the nearly four decades from 01/1981 to 09/2019. *Journal of Natural Products* 83(3): 770–803.
9. Pye, C. R., Bertin, M. J., Lokey, R. S., Gerwick, W. H. and Linington, R. G. (2017) Retrospective analysis of natural products provides insights for future discovery trends. *Proceedings of the National Academy of Sciences* 114(22): 5601–5606.
10. Quave, C. L. (2016) Antibiotics from nature: traditional medicine as a source of new solutions for combating antimicrobial resistance. *AMR Control*: July 13th, 2016. http://resistancecontrol.info/rd-innovation/antibiotics-from-nature-traditional-medicine-as-a-source-of-new-solutions-for-combating-antimicrobial-resistance/
11. Tu, Y. (2011) The discovery of artemisinin (qinghaosu) and gifts from Chinese medicine. *Nature Medicine* 17(10): 1217–1220.

## Abbreviations

HSA: highest single agent
NP: natural product

